# Dragon Fruit Peel (*Hylocereus undatus*) Modulates Hepatic Lipid Metabolism and Inflammation in a Rat Model of High-Fat, High-Fructose-Induced Metabolic Dysfunction

**DOI:** 10.3390/antiox14030319

**Published:** 2025-03-06

**Authors:** Siriwan Chumroenvidhayakul, Thavaree Thilavech, Mahinda Yapa Abeywardena, Michael Conlon, Julie Dallimore, Michael Adams, Beverly Muhlhausler, Sirichai Adisakwattana

**Affiliations:** 1 Center of Excellence in Phytochemical and Functional Food for Clinical Nutrition, Department of Nutrition and Dietetics, Faculty of Allied Health Science, Chulalongkorn University, Bangkok 10330, Thailand; siriwan.chu@kmitl.ac.th; 2 School of Food Industry, King Mongkut’s Institute of Technology Ladkrabang, Bangkok 10520, Thailand; 3 Department of Food Chemistry, Faculty of Pharmacy, Mahidol University, Bangkok 10400, Thailand; 4 CSIRO Health & Biosecurity, Kintore Avenue, Adelaide, SA 5000, Australia

**Keywords:** dragon fruit peel waste, high-fat high-fructose diet, insulin resistance, metabolic dysfunction, hepatic abnormalities

## Abstract

Metabolic dysfunction and hepatic abnormalities, such as those associated with high-fat, high-fructose (HFHFr) diets, are major contributors to obesity-related health issues. The growing interest in sustainable dietary interventions has highlighted the potential of plant-based byproducts. Dragon fruit (*Hylocereus undatus*) peel waste, rich in bioactive compounds such as dietary fibers, phenolics, and betacyanins, represents a promising functional ingredient for managing these disorders. This study investigated the effects of dragon fruit peel powder (DFP) on metabolic dysfunction and hepatic abnormalities induced by a HFHFr diet in rats. Over 12 weeks, the rats were fed a standard AIN-93M diet (control or C), C with 5% (*w*/*w*) DFP (C + DFP), a HFHFr diet, or a HFHFr diet with 5% (*w*/*w*) DFP (HFHFr + DFP). DFP supplementation significantly reduced HFHFr-induced body weight gain, visceral adiposity, insulin resistance, and dyslipidemia while also lowering systolic blood pressure and systemic oxidative stress markers. In the liver, DFP supplementation attenuated fat accumulation and lipid peroxidation, reduced glycogen storage abnormalities, and modulated the expression of lipid metabolism and inflammatory genes. These findings suggest that DFP may serve as a functional dietary supplement for preventing and managing metabolic disorders and liver abnormalities associated with excessive fat and fructose consumption.

## 1. Introduction

Excessive caloric intake, especially from diets high in saturated fats and simple carbohydrates such as sucrose, glucose, and fructose, combined with insufficient fiber consumption, has been linked to adverse metabolic outcomes [1]. Diets rich in fats and fructose, which are prevalent in Western populations, lead to metabolic disturbances, including hyperglycemia, insulin resistance, and dysregulated lipid metabolism [2]. These metabolic disturbances contribute to oxidative stress, inflammation, and impaired liver function, which are key mechanisms underlying the progression of metabolic dysfunction-associated steatotic liver disease (MASLD) [3]. Chronic low-grade systemic inflammation, induced by this dietary imbalance, disrupts insulin signaling pathways, promoting insulin resistance, dyslipidemia, and hepatic lipid accumulation, all of which contribute to liver damage [4]. In addition to lipid metabolism abnormalities, the consumption of high-fat, and high-fructose diets can cause the accumulation of glycogen in the liver, a condition known as glycogenosis. Recent studies suggest that glycogenosis, commonly observed in individuals with metabolic disorders, is linked to increased hepatic cellular injury and reduced levels of steatosis and fibrosis, highlighting its potential role in the progression of MASLD [5]. Given the rising prevalence of these metabolic conditions, effective interventions are needed, including lifestyle modifications such as increased physical activity and dietary changes [6].

Recent studies suggest that adherence to plant-based diets rich in fiber may offer protection against hepatic metabolic abnormalities. Arslanow et al. examined the effects of a short-term, low-calorie, high-fiber diet in patients with hepatic steatosis and found that increased dietary fiber intake could reduce hepatic fat content and improve metabolic syndrome parameters within 14 days [7]. Additionally, several studies have shown that plant bioactive compounds, particularly phytochemicals, may significantly reduce oxidative stress and inflammation, thereby mitigating the factors contributing to the progression of fatty liver disease [3,8]. A systematic review and meta-analysis of observational studies suggested that a higher intake of fruits and vegetables, due to their fiber and phytochemical content, is associated with a reduced risk of metabolic liver diseases [9].

Dragon fruit (*Hylocereus undatus*), commonly known as pitaya, has garnered considerable research interest due to its economic importance and potential health benefits [10]. In Thailand, dragon fruit pulp is widely consumed as fresh-cut fruit, snacks, and juice, owing to its high nutritional profile and reported anti-diabetic effects [11,12]. However, this consumption generates significant byproducts, primarily the peel, which constitutes approximately 33% of the fruit’s weight. The peel is often discarded, contributing to environmental pollution [10,11]. The emerging evidence suggests that dragon fruit peel waste is a valuable source of nutritional and phytochemical compounds. It contains abundant dietary fiber, pectin, phenolic acids, flavonoids, and betalains [10,11]. Particularly, the peel of *Hylocereus* spp. is a significant source of betalains, exhibiting higher concentrations than the flesh. These betalains possess strong antioxidant properties, suggesting the potential for the sustainable recovery of high-value substances from this agricultural byproduct [10,11]. Song et al. demonstrated that betacyanin extract (200 mg/kg body weight) significantly alleviated high-fat diet-induced insulin resistance, dyslipidemia, and hepatic lipid accumulation after 14 weeks of administration in C57BL/6 mice [13]. However, the mechanisms underlying the beneficial effects of dragon fruit peel on hepatic lipid metabolism and hepatocellular damage related to oxidative stress remain unclear. Moreover, most studies have investigated the biological effects of purified dragon fruit peel extract only in single-nutrient rich diet models, such as high-fat feeding [13,14]. The approach raises concern in the research of diet-induced metabolic dysregulation, as modern dietary lifestyles typically include high amounts of both saturated fat and simple sugars, particularly fructose [2,15].

The present study aimed to investigate the effects of dragon fruit peel powder (DFP) supplementation on metabolic dysfunction induced by a high-fat, high-fructose diet in rats. Specifically, this study also analyzed the impact of DFP on metabolic parameters, hepatic lipogenesis, and hepatocellular abnormalities. Through this investigation, the potential of DFP as a natural dietary intervention for alleviating metabolic disturbances associated with modern dietary lifestyles was evaluated.

## 2. Materials and Methods

### 2.1. Materials

Malondialdehyde (MDA), thiobarbituric acid (TBA), 2,4,6-Tripyridyl-S-Triazine (TPTZ), and sodium acetate trihydrate were obtained from Sigma-Aldrich (St. Louis, MO, USA). Trichloroacetic acid (TCA), hydrochloric acid (HCl), acetic acid, chloroform, and methanol were purchased from Merck (Darmstadt, Germany). DFP, derived from dragon fruit peel waste, was prepared according to a previously described method and stored at −20 °C until use [11]. The compositional analysis of DFP revealed 65.2% dietary fibers (including 15.6% soluble dietary fiber and 49.6% insoluble dietary fiber), 5.68% available carbohydrates, 6.37% protein, 15.91% ash, 5.81% moisture, and a trace amount of fat (1.06%). The total calories were 57.74 kcal/100 g. The total phenolic and total betacyanin contents in DFP were 454.79 ± 18.72 mg of gallic acid equivalent and 335.34 ± 2.26 mg of betanin equivalent/g powder, respectively [11].

### 2.2. Animals and Experimental Protocol

Male Sprague–Dawley rats (n = 32; 250–350 g at the start of the study; 8 weeks old) were housed in groups of two to three per cage, maintained in a climate-controlled room (22 ± 2 °C, 50–55% humidity) with a 12 h light/dark cycle. Male rats were selected based on their hormonal stability [16]. All procedures involving animals were conducted in accordance with the 8th edition (2013) of the Australian National Health & Medical Research Council code of practice for the care and use of animals for scientific purposes. The experimental protocol was approved by the Animal Ethics Committee of the University of Adelaide (Approval No.: S-2021-101). After a 2-week acclimatization period, the rats were provided ad libitum access to food and randomly assigned to one of four dietary groups (8 rats/group): (1) control group fed a standard AIN-93M diet (C); (2) control group supplemented with 5% (*w*/*w*) DFP (C + DFP); (3) high-fat, high-fructose diet group receiving 20% fat and 50% (*w*/*w*) fructose in the diet, with 10% (*v*/*v*) fructose in drinking water (HFHFr); and (4) HFHFr group supplemented with 5% (*w*/*w*) DFP (HFHFr + DFP). As DFP is naturally rich in dietary fiber and serves as an alternative fiber source, the concentration of DFP was chosen to provide an insoluble fiber level similar to the AIN-93M standard diet, which employs cellulose. The compositions and proximate analysis of the four experimental diets are provided in Tables S1 and S2 (Supplementary Materials), respectively. Daily monitoring of food consumption through weighing remaining food in hoppers before replenishment, and fluid intake measurement using calibrated cylinders for drinking water and 10% (*w/v*) fructose solution, enabled calculation of total caloric intake throughout the study period, with body weight measurements recorded at weekly intervals.

After 12 weeks, the rats were anesthetized with isoflurane and euthanized by exsanguination from the abdominal aorta. Non-fasting blood samples were collected from the abdominal aorta and stored at −80 °C for further analyses. Organs and visceral adipose tissues were dissected and weighed. Liver segments were sampled from multiple lobes of each animal and divided into two portions for histological analysis by immersing in 10% neutral buffered formalin. For gene expression analysis, a 20 mg sample of frozen liver tissue was preserved in cold RNAlater™ solution (Invitrogen™, Thermo Fisher Scientific, Waltham, MA, USA) (200 µL) and stored at −80 °C for 24 h prior to the experiment.

In this study, all biospecimens were collected from non-fasted rats, in accordance with animal welfare guidelines set by the ethics committee. To ensure controlled feed intake while minimizing inter-animal variation, food access was restricted for a defined period before euthanasia rather than implementing overnight fasting. This approach maintains scientific rigor while adhering to ethical standards for animal welfare.

### 2.3. Blood Pressure Measurement

During the 12-week experimental phase, systolic blood pressure in conscious animals was measured every 4 weeks using the tail-cuff method [17]. Before measurements began, the animals were acclimated to the Perspex tubes during two separate familiarization sessions. Measurements were conducted at 30 °C, with animals placed in the measuring tubes, and systolic blood pressure was recorded using an electro-sphygmomanometer combined with a pneumatic pulse transducer/amplifier (model 6m22931, six-channel NIBP system, Mediquip Pty Ltd., Loganholme, QLD, Australia) and BpMonWin Monitor version 1.33 software (IITC Life Science, Woodland Hills, CA, USA).

### 2.4. Biochemical Analysis

Plasma glucose concentrations and serum lipid profiles, including triglycerides (TG), total cholesterol (TC), high-density lipoprotein cholesterol (HDL-C), and low-density lipoprotein cholesterol (LDL-C), were determined using colorimetric enzymatic assay kits (Beckman Coulter, Ireland) following the manufacturer’s instructions. Serum insulin levels were quantified using a rat insulin enzyme-linked immunosorbent assay (ELISA) kit (Mercodia, Uppsala, Sweden).

To assess oxidative stress, plasma MDA levels, a biomarker of lipid peroxidation, were quantified using the thiobarbituric acid reactive substances (TBARS) assay, as described previously [18]. Briefly, 250 μL of plasma sample was mixed with 250 μL of 40% (*w*/*v*) trichloroacetic acid and centrifuged at 5500× *g* for 15 min at 4 °C to collect the supernatant. Subsequently, 500 μL of 0.67% (*w*/*v*) TBA solution was added to the supernatant and gently mixed. The mixture was heated at 95 °C for 10 min and then cooled to room temperature. Absorbance was measured at 530 nm, and MDA concentrations were calculated using MDA standard curve and expressed as μM MDA.

The total antioxidant capacity of plasma was determined using the ferric reducing ability of plasma (FRAP) assay, as described previously [19]. The protocol involved mixing 10 μL of plasma with 300 μL of freshly prepared and pre-warmed (37 °C) FRAP reagent, which consisted of 0.3 M sodium acetate buffer (pH 3.6), 10 mM TPTZ in 40 mM HCl, and 2.5 μL of 20 mM FeCl_3_ at a ratio of 10:1:1. The mixture was incubated for 5 min at 37 °C, and the absorbance was measured at 595 nm. The total antioxidant capacity was quantified using a standard curve of FeSO_4_ and expressed as μM FeSO_4_ equivalent.

### 2.5. Hepatic Lipid Accumulation and Oxidative Status Analysis

Total hepatic lipid content in rats was determined using a modified Bligh and Dyer method [20]. Briefly, individual rat liver samples (15 g) were homogenized in 100 mL of a chloroform/methanol/water mixture (2:2:1.8). The homogenate was then centrifuged at 3500× *g* for 10 min. The lipid-containing chloroform layer (lower phase) was carefully collected and evaporated overnight. The resulting residue was weighed, and the results were expressed as a percentage of lipid content relative to the initial liver tissue weight.

To assess oxidative stress in the hepatic tissue, lipid peroxidation was evaluated by measuring MDA levels using a TBARS assay kit (Sigma-Aldrich, St. Louis, MO, USA) according to the manufacturer’s instructions. Total protein content in the tissue samples was quantified by the Dumas combustion method using an Elementar rapid MAX N Exceed analyzer (Elementar, Hesse, Germany). The results were expressed as pmol MDA/mg protein.

### 2.6. Liver Histological Observation

Liver tissues were fixed in 10% (*v*/*v*) neutral-buffered formalin, dehydrated, and embedded in paraffin. The embedded tissues were sectioned to a thickness of 5 µm and stained with hematoxylin and eosin (H&E) for histological examination. The stained sections were examined under a light microscope (Leica DM200 LED, Wetzlar, Germany), and images were captured using a C-mount 0.55× lens and a digital camera (Leica MC170 HD, Zurich, Switzerland), with Leica Application Suite version 4.9.0 software. The pathological evaluation was conducted by an anatomical pathologist from the National Laboratory Animal Center, Salaya Campus, Mahidol University, Thailand. The diagnostic terms and glossary of incidental findings followed the guidelines set by the International Harmonization of Nomenclature and Diagnostic Criteria (INHAND). A four-level semi-quantitative scale was used to grade glycogen accumulation lesions: minimal (+1), mild (+2), moderate (+3), and severe (+4).

### 2.7. RNA Extraction and Quantitative Real-Time Polymerase Chain Reaction (qPCR) Analysis

Total RNA was extracted from the tissue using the ReliaPrep™RNA Miniprep Systems (Promega, Madison, WI, USA) following the manufacturer’s instructions. RNA concentration was quantified using a NanoDrop Lite Plus Spectrophotometer (Life Technology, Carlsbad, CA, USA). Subsequently, 1 µg of total RNA was reverse-transcribed into cDNA using GoScript™ reverse transcriptase (Promega, Madison, WI, USA) in a final volume of 20 µL. Quantitative real-time PCR (qRT-PCR) reactions were performed on the CFX Connect Real-Time PCR Detection System (Bio-Rad, Hercules, CA, USA) using iTaq™ Universal SYBR^®®^ Green Supermix and Commercial gene-specific PrimePCR™ PCR Primers (Bio-Rad, Hercules, CA, USA) for the following target genes: carbohydrate-responsive element-binding protein (*ChREBP*; Product No.: qRnoCED0001646), acetyl-CoA carboxylase (*Acaca*; Product No.: qRnoCID0001546), fatty acid synthase (*Fasn;* Product No.: qRnoCED0004370), diacylglycerol acyltransferase 2 (*Dgat2*; Product No.: qRnoCID0009232), peroxisome proliferator-activated receptor alpha (*Ppar*-α; Product No.: qRnoCID0004661), and carnitine palmitoyltransferase 1a (*Cpt1a*; Product No.: qRnoCID0009604). The inflammatory factors, including *TNF-α* (Forward: GAGCACGGAAAGCATGATCC; Reverse: TAGACAGAAGAGCGTGGTGG), *IL-1β* (Forward: GGGATGATGACGACCTGCTA; Reverse: TGTCGTTGCTTGTCTCTCCT), and *IL-6* (Forward: CTCATTCTGTCTCGAGCCCA; Reverse: TGAAGTAGGGAAGGCAGTGG), were also detected in liver tissue. The level of mRNA expression was calculated using the 2^−ΔΔCt^ method and normalized by the reference gene glyceraldehyde-3-phosphate dehydrogenase (*GAPDH*; Product No.: qRnoCID0057018). To validate the stability of *GAPDH* expression in liver tissue under the experimental conditions, one-way ANOVA was performed. The results indicated no significant differences in *GAPDH* expression between treatment groups (*p* = 0.868), confirming its suitability as a reference gene. The results were expressed as the level of relative quantity to control (normal diet).

### 2.8. Data Analysis

Data are presented as means ± standard error of the mean (SEM), with eight animals/group. The Shapiro–Wilk test was employed to assess the normality of data distribution. Based on the results, a two-way analysis of variance (ANOVA) was conducted to evaluate the effects of diet type, DFP supplementation, and their interaction, followed by posthoc Least Significant Difference (LSD) tests (SPSS Statistics 22, SPSS Inc., Chicago, IL, USA). The statistical analysis of the histological examination was performed using the Mann–Whitney U test. Statistical significance was established at *p* < 0.05.

## 3. Results and Discussion

### 3.1. Effects of DFP on Body Weight Gain, Food Intake, and Organ Weight

The rats fed a high-fat, high-fructose (HFHFr) diet exhibited a similar rate of body weight gain as the control rats on a normal diet (Table 1). However, supplementation with DFP resulted in a reduced body weight gain in both the normal and HFHFr diet groups. The results revealed a significant decrease in daily food intake among the rats fed the HFHFr diet compared to those on a normal diet, with the DFP reducing food intake only in the normal diet group. Additionally, the HFHFr group exhibited a significant increase in daily fluid intake; however, the addition of DFP to the HFHFr diet resulted in a significant reduction in daily fluid intake. When the caloric intake from both food and fluid was calculated, the total calorie intake was significantly higher in the HFHFr group compared to the normal diet group. However, the addition of DFP resulted in a reduced total calorie intake in both the HFHFr and normal diet groups.

Our findings revealed that the HFHFr diet increased fluid intake while reducing food consumption by 33.0% compared to the normal diet after 12 weeks. The increased fluid consumption in the rats fed the HFHFr diet is likely attributed to the appealing taste of fructose, which enhances palatability [15,21]. However, despite these changes, the rats on the HFHFr diet did not show a significant increase in body weight gain or visceral adiposity compared to the control rats. These results align with previous studies suggesting that reduced food intake in response to the high energy density of the HFHFr diet (5 kcal/g), compared to the standard diet (3.95 kcal/g), may act as a compensatory mechanism to regulate energy balance homeostasis and prevent excessive weight gain [15]. This compensatory response likely involves the integration of energy intake, central satiety signals, and energy metabolism via the autonomic nervous system, effectively limiting body weight gain and curbing visceral fat deposition, as reported in prior research [15]. In addition to this compensatory response, the physical characteristics of the HFHFr diet may have further contributed to the reduced solid food intake. Specifically, achieving a solid diet with a high concentration of fructose can be technically challenging and may result in a less palatable texture for the rats. This potential unpalatability could have also influenced food consumption and caloric intake [22].

Although no significant differences in initial and final body weights were observed among the groups, DFP supplementation effectively reduced body weight gain and visceral fat accumulation in both the normal and HFHFr diet groups. While DFP supplementation did not alter food intake in the HFHFr-fed rats, it significantly reduced fluid intake, leading to a notable decrease in total calorie consumption and particularly fructose, one of the key nutritional triggers of metabolic dysfunction. The beneficial effects of DFP may be attributed to its high fiber content (65.2%, with 15.6% soluble and 49.6% insoluble fiber) and hydration properties, as reported in our previous study [11]. Supplementation with 5% DFP increased the total and soluble dietary fiber by 1.30- and 2.27-fold in the control diet, and by 1.19- and 1.86-fold in the HFHFr diet, respectively (Table S2). Soluble fiber increases gut viscosity, slowing gastric emptying, while insoluble fiber accelerates transit time, reducing fructose absorption. Additionally, DFP increased the dietary moisture (Table S2), likely influencing fluid intake and contributing to adequate hydration despite a reduced beverage consumption [23]. These combined effects on reduced energy intake, absorption, and enhanced satiety likely contributed to body weight regulation, especially in the rats on the normal diet, as seen by the significant reductions in food and caloric intake.

An association between low fecal short-chain fatty acid (SCFA) concentrations, gut microbiota, and visceral obesity has been reported in humans [24]. Given that DFP provides soluble fiber, it may modulate the gut microbiome and enhance SCFA production, potentially explaining the reduction in visceral fat in both the normal and HFHFr diet groups. Further research is needed to explore DFP’s prebiotic potential in gut health.

### 3.2. Effects of DFP on Metabolic Parameters

As shown in Table 2, the rats fed the HFHFr diet exhibited significantly increased plasma glucose levels and serum insulin concentrations compared to the control rats on a normal diet. Supplementation with DFP in the HFHFr group effectively reversed these alterations, reducing blood glucose by 10.4% and serum insulin levels by 47.8%. Importantly, DFP administration did not further lower blood glucose in the control rats, nor did it affect their normal glucose metabolism.

The HFHFr diet significantly increased serum TG and TC levels while decreasing HDL-C levels compared to the control group (Table 2). Supplementation with DFP in HFHFr-treated rats reduced serum TG and TC concentrations to levels similar to those in the control rats fed a normal diet. Additionally, DFP attenuated the reduction of HDL-C levels in the HFHFr group. Notably, DFP treatment also significantly reduced serum LDL-C levels in both the HFHFr and control groups compared to their counterparts without DFP after 12 weeks.

The current study confirms that a 12-week consumption of a HFHFr diet, which is rich in fructose and saturated fat, induces metabolic disorders including insulin resistance and dyslipidemia. The underlying mechanism involves the excessive intake of fat and fructose, leading to elevated postprandial levels of TG, free fatty acids, glucose, and insulin levels, which trigger oxidative stress and an inflammatory response [3]. These alterations contribute to hepatic insulin resistance and increased lipogenesis [4]. The long-term consumption of the HFHFr diet further intensifies oxidative stress, exacerbates insulin resistance, and impairs glucose–lipid metabolism [4,25]. The lipid abnormalities associated with insulin resistance observed in this study are driven by high-fructose consumption and an overabundance of free fatty acids, which stimulate increased TG synthesis in the liver, leading to elevated serum TG [4,15]. This hypertriglyceridemia not only lowers HDL-C levels but also promotes the generation of small, dense LDL (sdLDL) particles from very-low-density lipoproteins (VLDL) [4]. The sdLDL particles are emerging as a significant CVD risk factor due to their contribution to endothelial dysfunction through multiple pathways, including the accumulation of oxidized LDL in arterial tissues, reduced nitric oxide (NO) production by the endothelium, and activation of the renin–angiotensin–aldosterone system [26].

Interestingly, supplementation with DFP for 12 weeks reversed several metabolic parameters altered by the HFHFr diet, restoring them to near-control levels. These findings are consistent with previous studies demonstrating that DFP’s bioactive compounds—including dietary fibers, betacyanins, and phenolic compounds—exert beneficial effects through multiple mechanisms. DFP’s soluble fiber plays a pivotal role in limiting fat and carbohydrate digestion and absorption and moderating postprandial responses [10,27]. This reduction in postprandial glycemic and lipidemic responses, combined with improvements in insulin resistance and hyperinsulinemia and a decreased substrate availability for de novo lipogenesis (DNL), likely contributes to the improved lipid profiles observed in the HFHFr-fed rats. Studies on red dragon fruit (*Hylocereus polyrhizus*) peels have shown that their soluble fiber content may ameliorate metabolic abnormalities by promoting beneficial gut microbiota growth and enhancing SCFA production [27]. SCFAs improve glycemia through binding to colonic G protein-coupled receptors, stimulating the release of appetite-regulating gut hormones, which influence glucose storage in muscle and adipose tissue [28]. Moreover, SCFAs enhance lipid metabolism by activating AMP-activated protein kinase (AMPK), which promotes fatty acid oxidation in liver and muscles, inhibits hepatic fatty acid synthesis, and reduces adipose tissue lipolysis [28]. Given the established link between gut dysbiosis and dyslipidemia, DFP’s soluble dietary fiber content could be responsible for the improvement of lipid profiles through microbiota modulation, contributing to decreased TG levels and increased HDL-C levels. Additionally, DFP’s soluble fiber also exhibits hypocholesterolemic effects via cholesterol and bile acid adsorption, promoting their fecal excretion and reducing serum cholesterol levels [29]. In addition to dietary fiber, the purified betacyanins, a subclass of betalains, derived from red dragon fruit have been shown to enhance adiponectin secretion and receptor expression in the adipocytes of mice fed a high-fat diet. This enhancement activates AMPK and peroxisome proliferator-activated receptor alpha (PPARα) signaling pathways, thereby improving fatty acid oxidation, glucose uptake, and overall insulin sensitivity [13]. Supplementation with betacyanins resulted in an increased relative abundance of the genus *Akkermansia*, suggesting that gut microbiota modulation is a mechanism through which betacyanins may protect against diet-induced metabolic disorders [14].

### 3.3. Effects of DFP on Plasma Oxidative Stress Status

After 12 weeks, the rats fed the HFHFr diet had significantly higher plasma levels of MDA, a marker of lipid peroxidation, compared to the control group. Supplementation with DFP in the HFHFr group significantly reduced MDA levels by 59.3% (Table 2). Regarding plasma antioxidant capacity, consumption of the HFHFr diet tended to reduce plasma FRAP values compared to the rats fed a normal diet. However, DFP supplementation led to a considerable increase in plasma FRAP levels: 4.9-fold greater in the C + DFP group and 3.2-fold greater in the HFHFr + DFP group.

DFP demonstrated strong antioxidant activity, as indicated by increased plasma FRAP levels and reduced plasma MDA levels in both the control and HFHFr-fed groups. This suggests that DFP supplementation mitigates oxidative stress while countering the pro-oxidative effects of fructose and uric acid, which are known contributors to endothelial damage [30].

### 3.4. Effects of DFP on Blood Pressure

**Figure 1:**
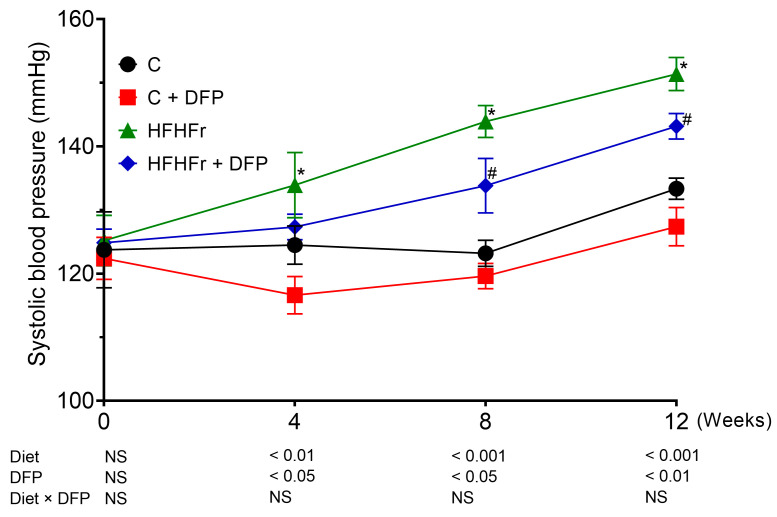
The effect of dragon fruit peel powder (DFP) on systolic blood pressure in normal diet (C), and high-fat, high-fructose diet (HFHFr) diet-fed rats. The results are expressed as mean ± SEM (*n* = 8). Two-way ANOVA revealed the effects of diet, DFP, and their interaction. Significant differences were determined using one-way ANOVA, followed by posthoc LSD tests; NS: non-significant differences. * *p* < 0.05 compared to C. # *p* < 0.05 compared to HFHFr.

Compared to the control group, the systolic blood pressure of the HFHFr-fed rats significantly increased by 8.73 and 18.72 mmHg after 4 and 8 weeks of HFHFr diet consumption, respectively. This increase continued, reaching 26.19 mmHg higher than the control group by the end of the 12-week study period. Supplementation with DFP in the HFHFr group effectively prevented this rise from week 4, resulting in significant reductions of 7.0% and 5.6% in systolic blood pressure at weeks 8 and 12, respectively (Figure 1). In contrast, DFP administration in the control rats did not affect blood pressure.

Studies in animal models have demonstrated that excessive fructose consumption contributes to the development of hypertension. This occurs through mechanisms including the conversion of fructose to uric acid, which reduces the availability of NO and induces endothelial dysfunction, as well as increased salt absorption and overstimulation of the sympathetic nervous system [31]. The reduction in elevated blood pressure with DFP supplementation may be due to its high betacyanin content, particularly betanin, which is a major dietary source of nitrates [13]. Similar to red beetroot, betanin may increase plasma nitrate levels, serving as a substrate for NO production. This enhances NO bioavailability, activates the cGMP pathway, and promotes vasodilation, resulting in a reduced systolic blood pressure [32]. Despite limited bioavailability due to gastrointestinal degradation, betacyanins can be absorbed through the small intestine, leading to systemic effects [33].

Supporting this, studies have shown that consuming betacyanin-rich beetroot significantly reduces systolic blood pressure in elderly individuals [34]. Similarly, betalain-rich supplements derived from red beetroot and prickly pear have been shown to alleviate hyperhomocysteinemia, a condition associated with endothelial cell damage and reduced vascular flexibility, thereby aiding in blood pressure reduction among coronary artery disease patients [35]. Furthermore, the dietary fiber content in DFP may help reduce hypertension in high-fructose diet models by delaying fructose digestion and slowing its absorption in the small intestine, which potentially reduces uric acid production [36]. Further research is needed to examine DFP’s effects on plasma uric acid, NO levels, the NO–cGMP pathway, and blood vessel changes, which will clarify how DFP mitigates high-fructose diet-induced hypertension.

### 3.5. Effects of DFP on Hepatic Lipid Accumulation, Oxidative Stress, and Histology

**Figure 2:**
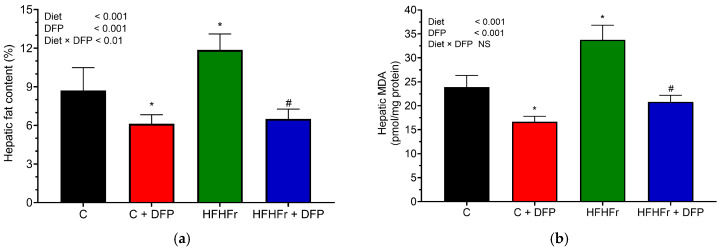
The effect of dragon fruit peel powder (DFP) on (**a**) hepatic lipid content and (**b**) hepatic malondialdehyde (MDA) levels in normal diet (C) and high-fat, high-fructose (HFHFr) diet-fed rats. The results are expressed as mean ± SEM (*n* = 8). Two-way ANOVA revealed the effects of diet, DFP, and their interaction. Significant differences were determined using one-way ANOVA, followed by posthoc LSD tests; NS: non-significant differences. * *p* < 0.05 compared to C. # *p* < 0.05 compared to HFHFr.

The HFHFr diet resulted in a significant increase in hepatic crude lipid accumulation, showing a 1.36-fold rise compared to the normal diet (*p* < 0.05) (Figure 2a). Notably, DFP supplementation significantly attenuated crude lipid accumulation in the livers of the HFHFr rats by 46%. The hepatic crude lipid content was also considerably reduced by 30% in the rats fed a normal diet when supplemented with DFP after the 12-week study period.

Furthermore, the MDA levels were assessed to evaluate lipid peroxidation and oxidative damage in liver tissue (Figure 2b). The results indicated that tissue MDA levels significantly increased by 1.5-fold in the HFHFr diet group compared to the control rats. Excessive intrahepatic lipid accumulation imposes a heavy burden on mitochondrial β-oxidation, leading to mitochondrial dysfunction and oxidative stress and contributing to lobular inflammation [3]. Consistent with previous studies, the HFHFr diet was associated with elevated hepatic MDA levels, which likely stimulate macrophage infiltration, promote inflammation, and result in hepatic stellate cell activation—key factors driving the progression of liver fibrosis, cirrhosis, and hepatocellular carcinoma [37]. DFP supplementation effectively reduced hepatic MDA levels by approximately 1.4-fold in the normal diet group and 1.6-fold in the HFHFr diet group. Clinical evidence has shown that antioxidant supplementation improves liver enzyme levels and histological outcomes in adults with nonalcoholic steatohepatitis [6]. In the present study, DFP treatment effectively reduced hepatic MDA concentrations, likely due to the presence of phenolic compounds and betacyanins in DFP. These compounds play a crucial role in the cellular defense against oxidative stress by scavenging ROS, thereby protecting hepatocyte cells from oxidative damage [10,11]. This reduction aligns with the findings of Vulić et al. (2014), who demonstrated that a beetroot pomace extract, rich in betacyanins and phenolic compounds, significantly decreased lipid peroxidation and increased antioxidant enzyme activity in a carbon tetrachloride-induced liver damage model [38].

**Figure 3:**
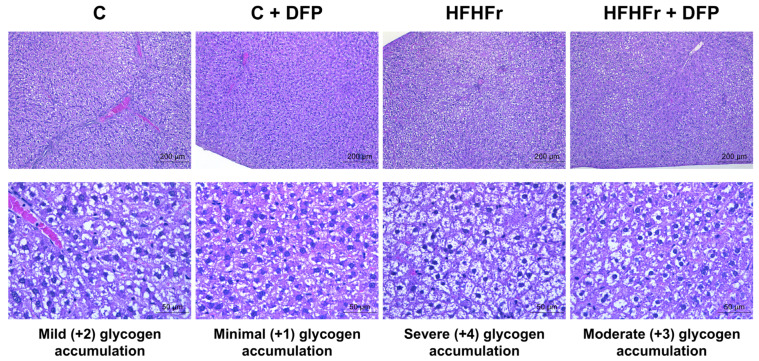
The effect of dragon fruit peel powder (DFP) on the hepatic glycogen accumulation in normal diet (C) and high-fat, high-fructose (HFHFr) diet-fed rats. Liver sections were stained with hematoxylin–eosin (10× magnification, 200 μm; 40× magnification, 50 μm).

The histological examination of the livers revealed diffuse glycogen accumulation in the hepatic parenchyma, characterized by poorly defined lacy clear cytoplasmic pallor and rarefaction, and centrally located nuclei observed across all treatment groups, exhibiting varying severity (Figure 3). Statistically significant differences were found in the severity of glycogen accumulation in the hepatocytes, which increased by 1.5-fold in the HFHFr group compared to the control group (Table 1). However, the inclusion of DFP in the diets did not result in a significant change in the progression of glycogenosis in rats fed either the control or HFHFr diet. The H&E-stained hepatic tissue showed limited evidence of steatohepatitis, with mild macrovesicular and microvesicular steatosis and lobular inflammation in 1–3 rats per group. Ballooning and fibrosis were absent (Figure S1), and no significant differences in non-alcoholic steatohepatitis (NASH) features or fibrosis staging were found among the groups.

The observed significant increase in hepatic glycogen accumulation in the HFHFr group aligns with previous studies on high-fructose diets in rodents [39,40]. Excessive fructose consumption contributes to insulin resistance, disrupts carbohydrate metabolism, and impairs gluconeogenesis regulation, leading to increased glycogen accumulation. This may serve as a compensatory mechanism to counteract fat accumulation and hyperglycemia. These findings are consistent with pathological observations in humans, where glycogenosis has been reported in up to 54% of MASLD cases, particularly in patients with type 2 diabetes and metabolic syndrome [5]. Notably, liver biopsies in these human cases have shown associations with increased hepatic cellular injury despite decreased steatosis and fibrosis [5]. These results highlight the need to further explore the dysregulation of carbohydrate and lipid metabolic pathways in insulin-resistant liver conditions.

### 3.6. Effects of DFP on Hepatic Gene Expression of Lipid Metabolism

**Figure 4:**
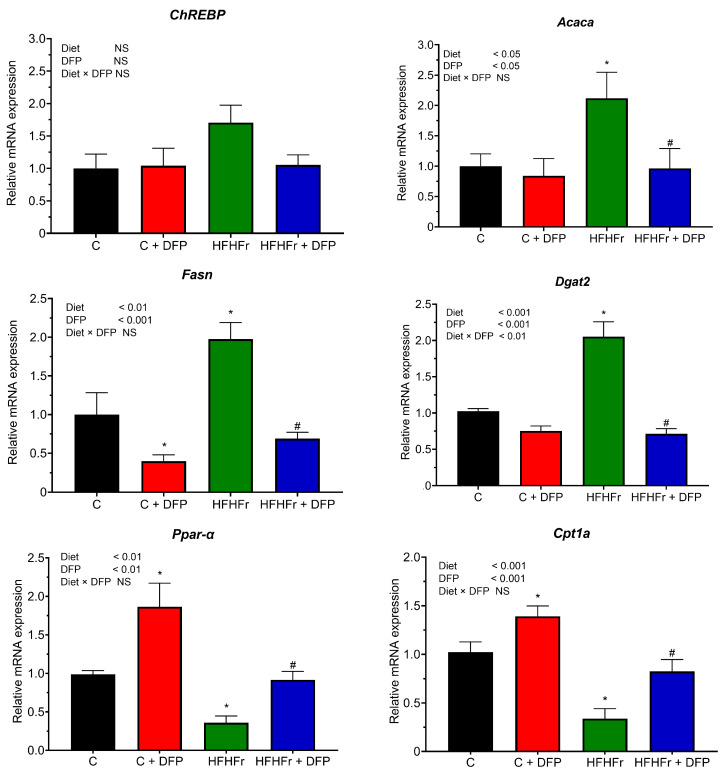
The effect of dragon fruit peel powder (DFP) on hepatic mRNA expression of lipid metabolism genes in normal diet (C) and high-fat, high-fructose (HFHFr) diet-fed rats. The results are expressed as mean ± SEM (*n* = 8). Two-way ANOVA revealed the effects of diet, DFP, and their interaction. Significant differences were determined using one-way ANOVA, followed by posthoc LSD tests; NS: non-significant differences. * *p* < 0.05 compared to C. # *p* < 0.05 compared to HFHFr.

The change in the mRNA expression of genes involved in fatty acid synthesis (*ChREBP*, *Acaca*, *Fasn*, and *Dgat2*) and fatty acid oxidation (*Ppar-α* and *Cpt1a*) were assessed after the 12-week study. As shown in Figure 4, the HFHFr diet significantly upregulated *Acaca* and *Dgat2* expression in hepatic tissue compared to the control group. However, after 12 weeks of dietary intervention, the HFHFr + DFP regimen reduced their expression to levels comparable to those in the control rats on a normal diet. Regarding fatty acid oxidation, DFP supplementation in the normal diet increased *Cpt1a* and *Ppar-α* expression by 1.75-fold and 1.87-fold, respectively. Furthermore, the HFHFr-induced downregulation of these genes in liver tissue was restored to normal levels with the DFP supplementation.

Prolonged excessive intake of a diet rich in fat and fructose is well-recognized as a key risk factor for the development of MASLD by promoting DNL in hepatocytes [4]. Our results demonstrated that a HFHFr diet tended to upregulate the expression of *ChREBP*, a major transcriptional regulator for hepatic lipogenesis, which is associated with various metabolic diseases and the severity of hepatic steatosis [41]. Consistent with previous studies, the mRNA levels of the essential enzymes in hepatic DNL, both *Acaca* and *Fasn*, were highest in the HFHFr group [4,42,43]. Additionally, after 12 weeks of HFHFr feeding, an upregulation of *Dgat2*, an enzyme responsible for TG synthesis, was observed in the hepatic tissue. Conversely, the HFHFr diet caused the downregulation of *Ppar-α* and *Cpt1a*, genes involved in fatty acid oxidation and cholesterol metabolism in the liver, resulting in increased intracellular hepatic cholesterol deposition, a key modulator of liver injury associated with NASH [42]. The changes in hepatic lipid metabolism gene expression may have contributed to the significant increase in serum total cholesterol and triglyceride levels observed after 12 weeks of the HFHFr diet. Previous studies have shown that high-fat, high-fructose diets suppress hepatic *Ppar-α* and *Cpt1a*, leading to marked steatosis and inflammation in the liver [43,44]. These factors contribute to hepatic lipid accumulation and the development of macro- and microvesicular steatosis.

Consuming DFP for 12 weeks nearly normalized the expression of hepatic fatty acid synthesis genes, including *ChREBP*, *Acaca*, *Fasn*, and *Dgat2*, while reestablishing *Ppar-α* and *Cpt1a* in rat livers. These mechanisms may explain the significant reduction in serum lipid profiles and hepatic fat content observed in both the DFP-supplemented rats on a normal diet and those on a HFHFr diet. It is likely that the fermentation of DFP by gut bacteria may produce SCFAs such as acetate and butyrate. These SCFAs activate the G-protein coupled receptors GPR41 and GPR43, stimulating AMPK activation and upregulating *Ppar-α*, thus promoting fatty acid oxidation [36]. Consistent with our results, a previous study using a purified betacyanin extract from DFP demonstrated the upregulation of fat utilization through β-oxidation via *Cpt1a* and *Cpt1b* while suppressing fatty acid synthesis by decreasing *Fasn* expression in high-fat diet-fed mice [13]. While reducing liver fat is generally beneficial, an excessive reduction in rats on a standard diet could disrupt normal physiological processes. Therefore, the long-term monitoring of liver function is crucial, especially in individuals with normal lipid profiles.

### 3.7. Effects of DFP on Hepatic Gene Expression of Pro-Inflammatory Cytokines

**Figure 5:**
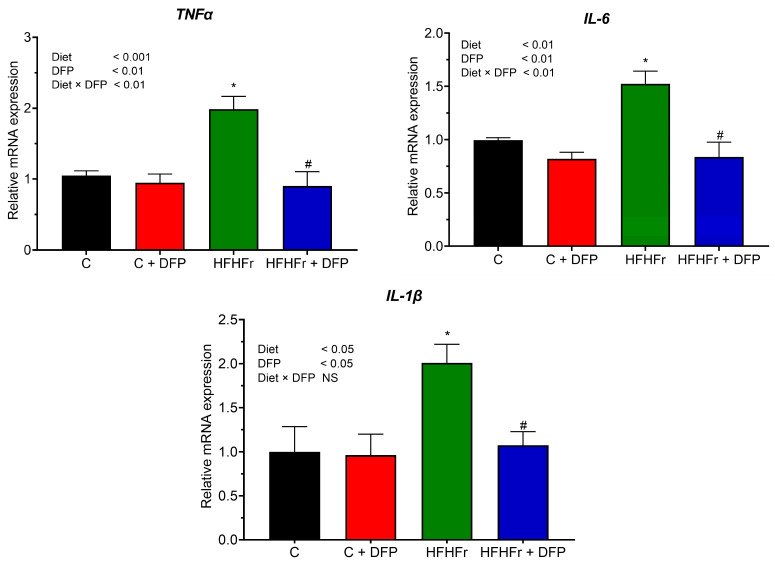
The effect of dragon fruit peel poweder (DFP) on hepatic mRNA expression of pro-inflammatory cytokines genes in normal diet (C) and high-fat, high-fructose diet (HFHFr) diet-fed rats. The results are expressed as mean ± SEM (*n* = 8). Two-way ANOVA revealed the effects of diet, DFP, and their interaction. Significant differences were determined using one-way ANOVA, followed by posthoc LSD tests; NS: non-significant differences. * *p* < 0.05 compared to C. # *p* < 0.05 compared to HFHFr.

The HFHFr diet resulted in a significant increase in inflammatory gene expression in hepatic tissue, showing a 2.0-fold increase in *TNF-α* and *IL-1β* and a 1.52-fold increase in *IL-6* compared to the control group (Figure 5). Supplementation with DFP in the HFHFr diet significantly suppressed the expression of these pro-inflammatory genes, restoring the levels to those observed in the control group. The most pronounced effect was seen for *TNF-α*, followed by *IL-1β* and *IL-6*, with mRNA expression reduced by 55%, 47%, and 41%, respectively. A similar reduction in these cytokines was observed in mice fed a high-fat diet supplemented with betacyanins from red pitaya fruit (200 mg/kg) for 14 weeks [14]. This reduction is likely attributed to the antioxidant and anti-inflammatory properties of DFP’s phenolic compounds and betacyanins, which may inhibit NF-κB activation, a central regulator of pro-inflammatory cytokine expression. Perhaps even more intriguing, betacyanin supplementation was shown to markedly increase the levels of *IL-10*, an anti-inflammatory cytokine that plays a protective role in reducing the severity of MASLD [3,14]. These anti-inflammatory effects are consistent with studies on other fruit byproducts. Specifically, citrus peel powder from *Jinggang* pomelo (*Citrus grandis* (L.) *Osbeck*) and *Gannan* navel orange (*Citrus sinensis Osbeck* cv. *Newhall*), rich in phenolic compounds, has been shown to significantly reduce *TNF-α* and *IL-6* levels in the livers of rats fed a high-fat diet after 12 weeks of supplementation [45].

Our findings demonstrate that DFP supplementation mitigated several metabolic disturbances and hepatic abnormalities associated with excessive fructose and saturated fat consumption, including significant reductions in diet-induced weight gain, dyslipidemia, and liver injury, along with improvements in insulin sensitivity, blood pressure regulation, and the modulation of lipid metabolism and inflammatory gene expression. To improve the accuracy of gene expression analysis, future studies should use multiple validated reference genes. This study’s use of only male rats minimized the variability due to hormonal fluctuations but limited the generalizability of our findings to females, who may respond differently due to sex differences in metabolic syndrome. Male rats may be more susceptible to certain metabolic dysfunctions, such as lipid metabolism and insulin resistance. Therefore, future research should include females for a more comprehensive understanding of DFP’s effects. To validate these promising findings, clinical trials are needed to assess the impact of DFP supplementation on human health. The feasibility of incorporating DFP as a functional ingredient into various food products has already been demonstrated, with successful additions to cookies, beef burgers, and instant noodles at concentrations ranging from 1–6% [46,47,48]. These findings underscore the potential of DFP as a dietary supplement warranting further investigation.

## 4. Conclusions

This study demonstrated that the whole DFP provided the synergistic effects of its phytochemicals and dietary fiber content to effectively mitigate the adverse effects of diet-induced metabolic dysfunctions. DFP intake improved glucose metabolism, enhanced lipid profiles, and positively influenced the systolic blood pressure. Additionally, DFP exhibited significant antioxidant activity by reducing systemic oxidative stress markers. It also protected against HFHFr diet-induced liver abnormalities by decreasing the fat content and lipid peroxidation while potentially reducing glycogen accumulation in the liver tissue. These effects were associated with the downregulation of lipogenic and pro-inflammatory cytokine genes along with the upregulation of genes involved in lipid β-oxidation. These findings suggest that DFP has potential as a functional food ingredient for preventing and managing metabolic disorders and liver abnormalities.

## Figures and Tables

**Table 1:** The effect of dragon fruit peel (DFP) on body weight gain, food intake, liver weight, visceral fat tissue, and glycogen accumulation levels in hepatocytes in normal diet (C) and high-fat, high-fructose (HFHFr) diet-fed rats.

Parameters	Experimental Groups				Significance of Effects		
	C	C + DFP	HFHFr	HFHFr + DFP	Diet	DFP	Diet × DFP
Initial body weight (g)	334.5 ± 14.2	329.3 ± 14.5	339.5 ± 12.3	338.6 ± 14.2	NS	NS	NS
Final body weight (g)	698.9 ± 25.2	677.7 ± 29.5	679.9 ± 20.1	642.6 ± 22.2	NS	NS	NS
Body weight gain (g)	329.8 ± 24.2	279.3 ± 18.9 *	303.8 ± 11.4	248.5 ± 12.2 ^#^	NS	<0.01	NS
Food intake (g/rat/day)	31.2 ± 0.6	28.1 ± 0.3 *	20.9 ± 0.5 *	19.9 ± 0.4	<0.001	<0.001	<0.05
Caloric intake from food (kcal/rat/day)	123.5 ± 2.4	109.6 ± 1.3 *	142.5 ± 2.3 *	130.1 ± 2.6 ^#^	<0.001	<0.001	NS
Fluid intake (mL/rat/day)	37.7 ± 1.1	33.5 ± 0.3	100.2 ± 3.0 *	81.1 ± 3.5 ^#^	<0.001	<0.001	<0.01
Caloric intake from fluid (kcal/rat/day)	ND	ND	38.8 ± 1.5 *	34.0 ± 1.6 ^#^	<0.001	<0.05	<0.05
Calorie intake (kcal/rat/day)	123.5 ± 2.4	109.6 ± 1.3 *	142.5 ± 2.3 *	130.1 ± 2.6 ^#^	<0.001	<0.001	NS
Visceral fat tissue (g)	43.6 ± 2.4	31.0 ± 2.0 *	46.7 ± 2.3	33.1 ± 2.9 ^#^	NS	<0.001	NS
Visceral fat tissue to body weight ratio (%)	7.6 ± 0.1	4.4 ± 0.3 *	6.7 ± 0.5	4.7 ± 0.4 ^#^	NS	<0.001	NS
Liver (g)	22.5 ± 0.6	18.1 ± 0.7	27.0 ± 0.9 *	21.0 ± 0.7 ^#^	<0.001	<0.001	NS
Liver to body weight ratio (%)	3.2 ± 0.2	2.7 ± 0.1	4.0 ± 0.2 *	3.2 ± 0.1 ^#^	<0.001	<0.01	NS
Glycogen accumulation	2.4 ± 0.7	2.2 ± 0.6	3.6 ± 1.02 *	3.1 ± 0.7	-	-	-

The results are expressed as mean ± SEM (*n* = 8). Two-way ANOVA revealed the effects of diet, DFP, and their interaction. The same row indicates significant difference at *p* < 0.05 by one-way ANOVA, followed by posthoc LSD tests; NS: non-significant differences. The Mann–Whitney U test was performed for statistical analysis of the glycogen accumulation. * *p* < 0.05 compared to C. ^#^ *p* < 0.05 compared to HFHFr.

**Table 2:** The effect of dragon fruit peel powder (DFP) on metabolic parameters in normal diet (C) and high-fat, high-fructose (HFHFr) diet-fed rats.

Parameters	Experimental Groups				Significance of Effects			
	C	C + DFP	HFHFr	HFHFr + DFP	Diet	DFP	Diet × DFP	
Plasma glucose (mmol/L)	9.22 ± 0.16	9.08 ± 0.17	10.07 ± 0.21 *	9.02 ± 0.30 ^#^	NS	<0.01	NS	
Serum insulin (μg/L)	1.13 ± 0.13	0.96 ± 0.10	1.84 ± 0.25 *	0.96 ± 0.08 ^#^	<0.05	<0.01	<0.05	
Serum TC (mmol/L)	2.00 ± 0.12	1.77 ± 0.07	2.66 ± 0.18 *	2.24 ± 0.12 ^#^	<0.001	<0.01	NS	
Serum LDL-C (mmol/L)	0.60 ± 0.04	0.42 ± 0.02 *	0.61 ± 0.03	0.51 ± 0.03 ^#^	NS	<0.01	NS	
Serum HDL-C (mmol/L)	1.26 ± 0.07	1.50 ± 0.10	0.91 ± 0.02 *	0.99 ± 0.04 ^#^	<0.001	<0.01	NS	
Serum TG (mmol/L)	1.54 ± 0.27	1.21 ± 0.17	3.83 ± 0.36 *	1.89 ± 0.17 ^#^	<0.001	<0.001	<0.01	
Plasma MDA (μM MDA)	2.94 ± 0.02	2.83 ± 0.03 *	8.97 ± 0.06 *	3.65 ± 0.02 ^#^	<0.001	<0.001	<0.001	
Plasma FRAP (μM FeSO_4_)	163.35 ± 7.36	807.50 ± 10.95 *	130.14 ± 8.29	417.63 ± 30.92 ^#^	<0.001	<0.001	<0.001	

The results are expressed as mean ± SEM (*n* = 8). Two-way ANOVA revealed the effects of diet, DFP, and their interaction. The same row indicates significant difference at *p* < 0.05 by one-way ANOVA, followed by posthoc LSD tests; NS: non-significant differences. * *p* < 0.05 compared to C. ^#^ *p* < 0.05 compared to HFHFr. TG—triglycerides; TC—total cholesterol; HDL-C—high-density lipoprotein cholesterol; LDL-C—low-density lipoprotein cholesterol; MDA—malondialdehyde; FRAP—ferric reducing ability of plasma.

## Data Availability

Data are contained within the article or Supplementary Materials.

## Supplementary Materials

The following supporting information can be downloaded at: https://www.mdpi.com/article/10.3390/antiox14030319/s1, Table S1. Composition of experimental diets; Table S2. Proximate composition of experimental diets; Figure S1. The effect of dragon fruit peel (DFP) on the liver histopathology in normal diet (C), and high-fat, high-fructose diet (HFHFr) diet-fed rats. Liver sections were stained with hematoxylin-eosin (20× magnification, 100 μm; 40× magnification, 50 μm). Black arrows indicate the presence of macrovesicular steatosis; blue arrows indicate the presence of microvesicular steatosis; circle indicates inflammatory infiltrate.
