# Supplementary material for: Dragon Fruit Peel (Hylocereus undatus) Modulates Hepatic Lipid Metabolism and Inflammation in a Rat Model of High-Fat, High-Fructose-Induced Metabolic Dysfunction
Source: Antioxidants (Basel). 2025 Mar 6;14(3):319. doi: 10.3390/antiox14030319 (PMC11939235; doi:10.3390/antiox14030319)
Supplement: Supplementary file 1 [file antioxidants-14-00319-s001.zip › antioxidants-3483921-supplementary.pdf]

Dragon Fruit Peel (*Hylocereus undatus*) Modulates Hepatic Lipid Metabolism and Inflammation in a Rat Model of High-Fat, High-Fructose-Induced Metabolic Dysfunction

Siriwan Chumroenvidhayakul [1,2], Thavaree Thilavech [3,*], Mahinda Yapa Abeywardena [4], Michael Conlon [4], Julie Dallimore [4], Michael Adams [4], Beverly Muhlhausler [4] and Sirichai Adisakwattana [1,*]

[1] Center of Excellence in Phytochemical and Functional Food for Clinical Nutrition, Department of Nutrition and Dietetics, Faculty of Allied Health Science, Chulalongkorn University, Bangkok 10330, Thailand; siriwan.chu@kmitl.ac.th
[2] School of Food Industry, King Mongkut's Institute of Technology Ladkrabang, Bangkok 10520, Thailand
[3] Department of Food Chemistry, Faculty of Pharmacy, Mahidol University, Bangkok 10400, Thailand
[4] CSIRO Health & Biosecurity, Kintore Avenue, Adelaide, SA 5000, Australia; mahinda.abeywardena@csiro.au (M.Y.A.); michael.conlon@csiro.au (M.C.); julie.dallimore@csiro.au (J.D.); michael.adams@csiro.au (M.A.); beverly.muhlhausler@csiro.au (B.M.)
* Correspondence: thavaree.thi@mahidol.ac.th (T.T.); sirichai.a@chula.ac.th (S.A.)

Table S1. Composition of experimental diets

Diet composition (%)	Experimental groups			
	C	C + DFP	HFHFr	HFHFr + DFP
Corn starch	53.2	51.8	0	0
Sucrose	10	10	0	0
Fructose	0	0	50	48.8
Casein	20	20	20	20
Sunflower seed oil	7	7	2.5	2.5
Lard	0	0	17.5	17.5
Mineral mixture	4	4	4	4
Vitamin mixture	1	1	1	1
Tert-Butylhydroquinone	0.0014	0.0014	0.0014	0.0014
Choline	0.25	0.25	0.25	0.25
Cellulose	5	1.7	5	1.7
DFP	0	5	0	5
Fructose in drinking water (*w/v*)	0	0	10	10

C: normal diet (AIN 93M); C + DFP: normal diet with 5% (*w/w*) dragon fruit peel powder; HFHFr: high-fat, high-fructose diet; HFHFr + DFP: high-fat, high-fructose diet with 5% (*w/w*) dragon fruit peel powder.

Table S2. Proximate composition of experimental diets

Composition	Experimental groups			
	C	C + DFP	HFHFr	HFHFr + DFP
Total carbohydrate (g/100 g)	72.2	66.2	57.3	56.6
Starch (g/100 g)	53.4	46.2	0.3	0.9
Sugar (g/100 g)	12	11.8	50.8	48.3
Total dietary fiber (g/100 g)	6.3	8.2	6.2	7.4
Soluble dietary fiber (g/100 g)	1.5	3.4	1.4	2.6
Insoluble dietary fiber (g/100 g)	4.8	4.8	4.8	4.8
Protein (g/100 g)	17.3	17	16.9	16.3
Fat (g/100 g)	7.2	6.9	18.7	17.5
Moisture (g/100 g)	4.3	7.1	5.1	6.7
Ash (g/100 g)	2.9	3.5	2.6	3.8

C: normal diet (AIN 93M); C + DFP: normal diet with 5% (*w/w*) dragon fruit peel powder; HFHFr: high-fat, high-fructose diet; HFHFr + DFP: high-fat, high-fructose diet with 5% (*w/w*) dragon fruit peel powder**.**

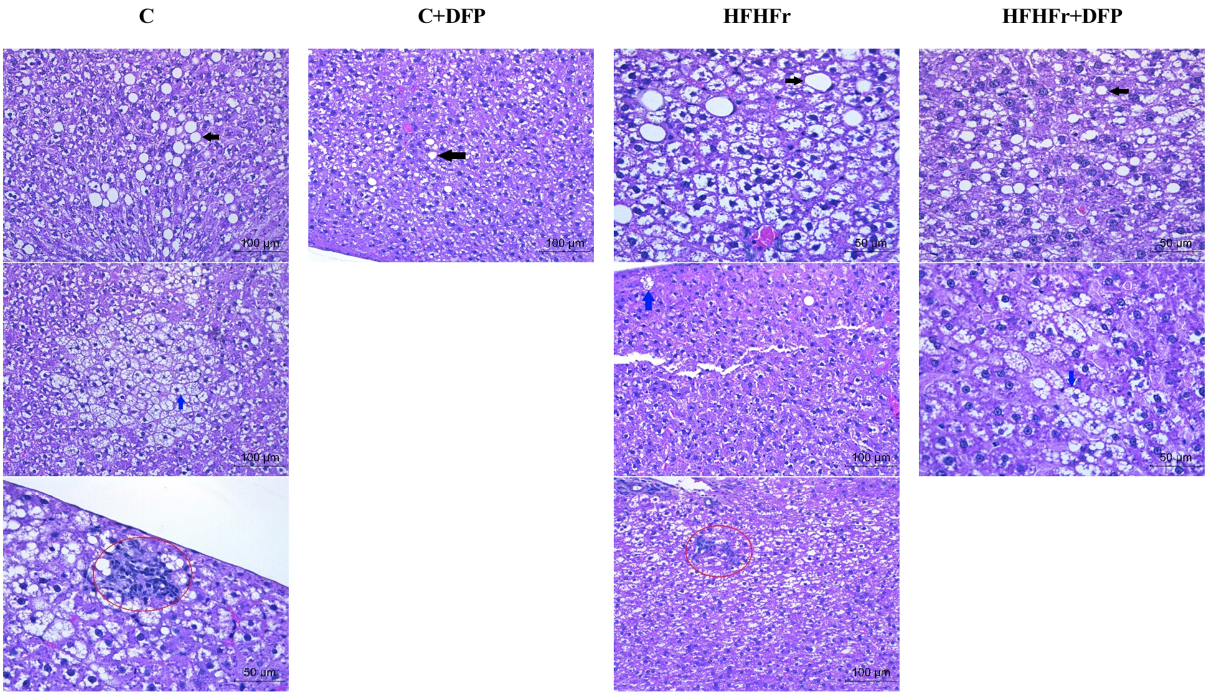

Figure S1. The effect of dragon fruit peel (DFP) on the liver histopathology in normal diet (C), and high-fat, high-fructose diet (HFHFr) diet-fed rats. Liver sections were stained with hematoxylin-eosin (20× magnification, 100 µm; 40× magnification, 50 µm). Black arrows indicate the presence of macrovesicular steatosis; blue arrows indicate the presence of microvesicular steatosis; circle indicates inflammatory infiltrate.
